# Opening the door to a new era: A dual‐chamber leadless pacemaker

**DOI:** 10.1002/joa3.70100

**Published:** 2025-05-28

**Authors:** Miyo Nakano, Yusuke Kondo, Richard H. Hongo, Hiroyuki Takaoka, Yoshio Kobayashi

**Affiliations:** ^1^ Department of Cardiovascular Medicine Chiba University Graduate School of Medicine Chiba Japan; ^2^ Atrial Fibrillation & Complex Arrhythmia Center Sutter Pacific Medical Foundation California Pacific Medical Center San Francisco San Francisco CA USA

**Keywords:** Aveir, dual‐chamber, leadless pacemaker

## Abstract

Dual‐chamber leadless pacing with Aveir DR offers a breakthrough alternative for patients at high risk of infection or with limited vascular access. By enabling both atrial and ventricular pacing without transvenous leads, this technology improves AV synchrony and may revolutionize future cardiac rhythm management, including defibrillation and resynchronization therapies.

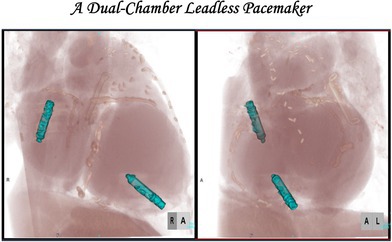

A 65‐year‐old woman presented signs of heart failure after the implantation of a leadless pacemaker. Seven years earlier, she had undergone transvenous dual‐chamber pacemaker implantation for sick sinus syndrome. However, 4 months ago, she developed a pacemaker‐related infection, leading to septic shock, and underwent complete device removal.

During hospitalization, a coronary angiogram revealed three‐vessel coronary artery disease, including the left main artery. After antibiotic treatment, she underwent coronary artery bypass grafting. Additionally, she had a history of paroxysmal atrial fibrillation. Furthermore, the patient had a history of Sjögren's syndrome, IgA nephropathy, and chronic kidney disease, placing her at high risk for recurrent infections. Given these considerations, a leadless pacemaker (Aveir VR) was chosen instead of a transvenous system to minimize infection risk. However, after the implantation of the leadless pacemaker, her heart failure worsened, which was attributed to frequent ventricular pacing (Vp). Electrocardiographic and transthoracic echocardiographic studies revealed atrioventricular dyssynchrony contributing to the exacerbation of heart failure. Atrial pacing (Ap) was deemed necessary to maintain physiological atrioventricular synchrony. To mitigate the risk of infection and lead‐related complications, a leadless atrial pacemaker (Aveir AR) was implanted, and the pacing mode was upgraded to DDD mode (Figure 1A,B).

**FIGURE 1:**
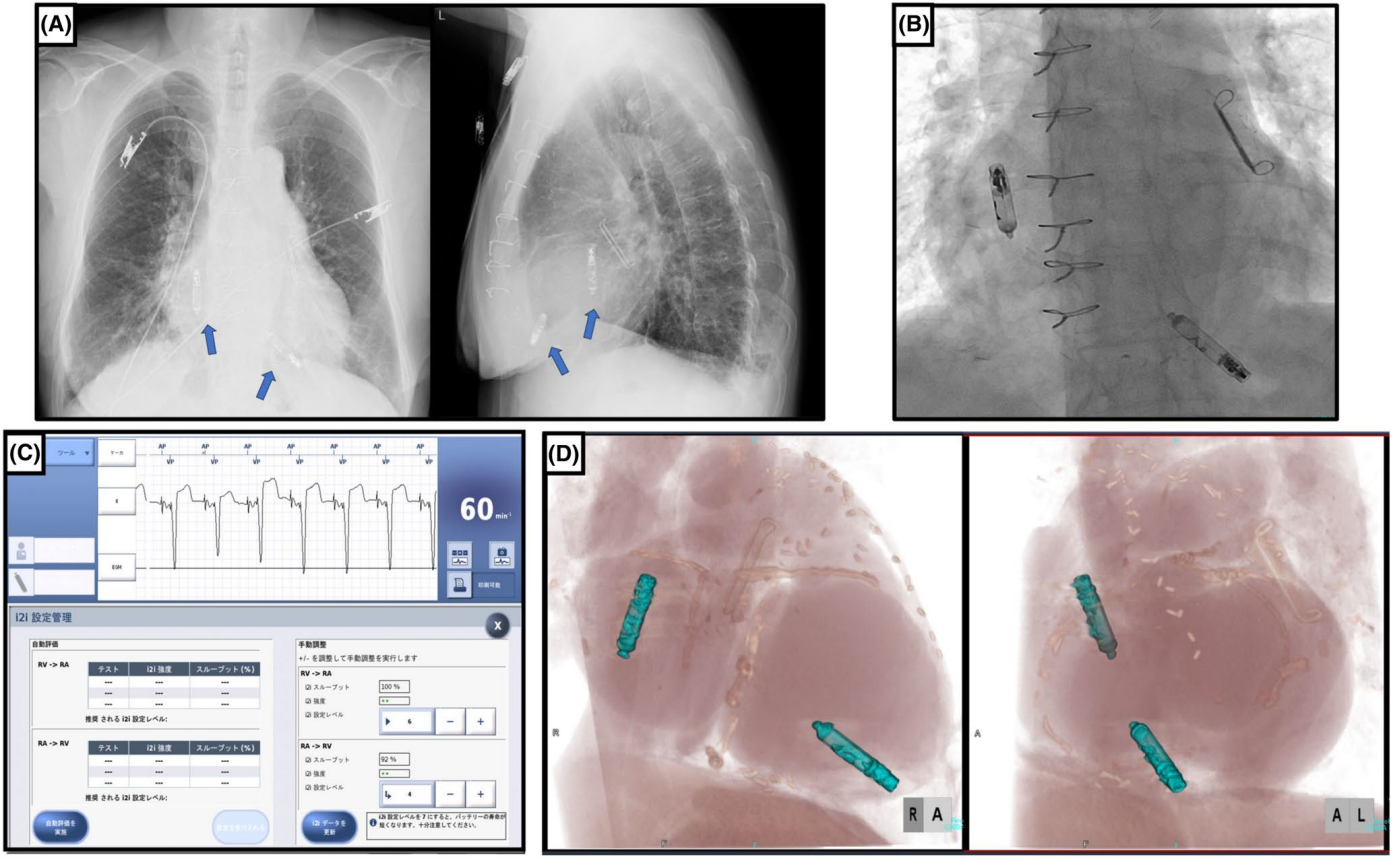
(A) Two‐view chest x‐ray of dual‐chamber leadless pacemaker. (B) Fluoroscopy image confirming successful implantation of Aveir AR and DR devices. (C) Intracardiac electrograms from the Aveir DR system showing appropriate sensing and pacing function. (D) Computed tomography image showing correct device positioning.

Device interrogation confirmed stable sensing, pacing thresholds, and impedance values (threshold of 1.75 V@0.4 ms, 1.25 V@1.5 ms), ensuring proper device function (Figure 1C). Computed tomography imaging showed no complications such as thrombus formation, perforation, or device dislodgment, confirming that the device was appropriately positioned on the posterior wall at the base of the right atrial appendage (Figure 1D). Serial echocardiographic evaluations demonstrated gradual improvement in cardiac function, with the left ventricular ejection fraction increasing from 40% to 47% and the E/A ratio improving from unmeasurable to 2.0. Following the initiation of atrial pacing, clinical assessments also indicated alleviation of heart failure symptoms, allowing for a reduction in the dosage of diuretics. The patient tolerated the procedure well, experienced no perioperative complications, and was discharged in stable condition. One of the limitations of DDD mode is its relatively short battery life. However, in the present case, DDD mode was initially selected to ensure atrioventricular synchrony and stabilize heart failure. We plan to continue DDD pacing during the acute phase, and once the heart failure symptoms improve, we will consider switching to AAI mode with VVI backup to optimize battery longevity.

This case highlights the utility of leadless dual‐chamber pacing in patients at high risk of infection and pacing‐induced heart failure due to atrioventricular dyssynchrony. The addition of a leadless atrial pacemaker effectively alleviated heart failure symptoms while avoiding the risks associated with transvenous leads. The patient remained stable postoperatively and continued to improve during follow‐up.

In Japan, the Micra leadless pacemaker became available for clinical use in 2017, and its minimally invasive nature and reduced risk of infection have contributed to its rapid adoption, particularly in patients at high risk of infection or with limited vascular access.^1^, ^2^ Subsequently, Aveir DR was approved for insurance reimbursement in February 2025. Micra AV provides atrioventricular synchrony by sensing atrial mechanical activity; however, it does not support atrial pacing. In contrast, Aveir DR enables true dual‐chamber pacing through separate atrial and ventricular devices, allowing both sensing and pacing in each chamber. Aveir DR, reported as the world's first dual‐chamber leadless pacemaker, addresses key limitations of traditional transvenous systems while enabling physiological atrioventricular synchrony, a major clinical advantage.^3^ With innovative technology enabling wireless communication between the atrium and ventricle, further advancements are expected to expand its indications. In conclusion, this case illustrates how dual‐chamber leadless pacing offers an effective solution for complex patients, particularly those with a history of infection or comorbidities that limit the use of traditional pacing systems. Looking ahead, the development of leadless technology may extend beyond pacemakers to implantable cardioverter‐defibrillators and cardiac resynchronization therapy devices, marking the opening of a new era in cardiac rhythm management.

## CONFLICT OF INTEREST STATEMENT

Dr. Kondo received lecture fees from Daiichi‐Sankyo, Abbott Medical Japan, Biotronik Japan, Boston Scientific, Japan Lifeline, Medtronic, and research funds from Daiichi‐Sankyo and Boston Scientific. Dr. Nakano belongs to the endowed department from Abbott Medical Japan, Biotronik Japan, and Fukuda Denshi. Dr. Kobayashi received lecture fees from Abbott Medical Japan, Bayer Japan, Bristol‐Myers Squibb, Boehringer Ingelheim, Daiichi‐Sankyo, and scholarship funds from Takeda Pharmaceutical, Abbott Medical Japan, Terumo, Otsuka Pharmaceutical, Boehringer Ingelheim, Astellas, Daiichi‐Sankyo, Win International, Japan Lifeline, and Nipro.

## STATEMENTS RELATING TO OUR ETHICS AND INTEGRITY POLICIES

The consent of the publication was obtained.
